# Low Socioeconomic Status Is Associated with Prolonged Times to Assessment and Treatment, Sepsis and Infectious Death in Pediatric Fever in El Salvador

**DOI:** 10.1371/journal.pone.0043639

**Published:** 2012-08-22

**Authors:** Ronald Gavidia, Soad L. Fuentes, Roberto Vasquez, Miguel Bonilla, Marie-Chantal Ethier, Caroline Diorio, Miguela Caniza, Scott C. Howard, Lillian Sung

**Affiliations:** 1 Pediatric Oncology, Benjamin Bloom National Children’s Hospital, San Salvador, El Salvador; 2 Child Health Evaluative Sciences, The Hospital for Sick Children, Toronto, Canada; 3 Division of Haematology/Oncology, The Hospital for Sick Children, Toronto, Canada; 4 Department of Infectious Diseases, St. Jude Children's Research Hospital, Memphis, Tennessee, United States of America; 5 Department of Oncology, St. Jude Children's Research Hospital, Memphis, Tennessee, United States of America; 6 International Outreach Program, St. Jude Children's Research Hospital, Memphis, Tennessee, United States of America; Indiana University, United States of America

## Abstract

**Background:**

Infection remains the most common cause of death from toxicity in children with cancer in low- and middle-income countries. Rapid administration of antibiotics when fever develops can prevent progression to sepsis and shock, and serves as an important indicator of the quality of care in children with acute lymphoblastic leukemia and acute myeloid leukemia. We analyzed factors associated with (1) Longer times from fever onset to hospital presentation/antibiotic treatment and (2) Sepsis and infection-related mortality.

**Method:**

This prospective cohort study included children aged 0–16 years with newly diagnosed acute leukemia treated at Benjamin Bloom Hospital, San Salvador. We interviewed parents/caregivers within one month of diagnosis and at the onset of each new febrile episode. Times from initial fever to first antibiotic administration and occurrence of sepsis and infection-related mortality were documented.

**Findings:**

Of 251 children enrolled, 215 had acute lymphoblastic leukemia (85.7%). Among 269 outpatient febrile episodes, median times from fever to deciding to seek medical care was 10.0 hours (interquartile range [IQR] 5.0–20.0), and from decision to seek care to first hospital visit was 1.8 hours (IQR 1.0–3.0). Forty-seven (17.5%) patients developed sepsis and 7 (2.6%) died of infection. Maternal illiteracy was associated with longer time from fever to decision to seek care (P = 0.029) and sepsis (odds ratio [OR] 3.06, 95% confidence interval [CI] 1.09–8.63; P = 0.034). More infectious deaths occurred in those with longer travel time to hospital (OR 1.36, 95% CI 1.03–1.81; P = 0.031) and in families with an annual household income <US$2,000 (OR 13.90, 95% CI 1.62–119.10; P = 0.016).

**Interpretation:**

Illiteracy, poverty, and longer travel times are associated with delays in assessment and treatment of fever and with sepsis and infectious mortality in pediatric leukemia. Providing additional education to high-risk families and staying at a nearby guest house during periods of neutropenia may decrease sepsis and infectious mortality.

## Background

There has been steady improvement in treatment outcomes for children with acute leukemia over the past few decades. Unfortunately, these advances in survival have not fully translated into low- and middle-income countries (LMIC) where event-free survival is significantly lower than in high-income countries [1] because of higher rates of relapse, abandonment of treatment, and treatment-related mortality (TRM).

In El Salvador, TRM is responsible for about 50% of deaths in children with acute lymphoblastic leukemia (ALL) and acute myeloid leukemia (AML), [2] with a two-year cumulative incidence of TRM of 12.5±1.7% for ALL and 35.1±6.4% for AML (P<0.0001). Infections were the most common cause of TRM, and 12.3% of episodes of febrile neutropenia resulted in death. [3] Among children with ALL (but not AML), low monthly income and low parental education were associated with significantly higher TRM. [2] Given that ALL is primarily managed in the outpatient setting and that AML is primarily managed in the inpatient setting, we hypothesized that delays in seeking care for febrile neutropenia may be the link between socioeconomic status and TRM in ALL and might explain the absence of a connection in children with AML, who remain in or near the hospital during their entire course of treatment.

In this study, we prospectively evaluated the association between socioeconomic status and times to assessment and treatment for fever, and with rates of sepsis and infectious mortality.

## Methods

### Participants and Setting

We included children younger than 17 years with ALL and AML newly diagnosed from May 1, 2008 to March 21, 2011 and treated at Benjamin Bloom National Children’s Hospital (Hospital Bloom) in San Salvador, El Salvador. Hospital Bloom has had a long standing partnership with St. Jude Children’s Research Hospital. We excluded those with an initial palliative intent of treatment. Hospital Bloom is the only hospital in El Salvador that admits and treats children with cancer, and cares for approximately 200 newly diagnosed children with cancer each year. Treatment was provided at no cost to families; accommodation and child care were also offered free to families living significant distances from the hospital to reduce abandonment of therapy. This study was approved by the Institutional Review Boards at Hospital Bloom and The Hospital for Sick Children, Toronto, Canada and informed written consent was obtained from parents, children ages 12 to 18 and healthcare professionals.

**Table 1:** Demographics of Enrolled Children with Newly Diagnosed Acute Lymphoblastic Leukemia and Acute Myeloid Leukemia (N = 251).

Characteristic	Value
**Child Characteristics**	
Male (%)	133 (53.0)
Median age in years (IQR)	5.2 (2.8, 9.2)
Median BMI percentile (IQR)^a^ (n = 218)	44.9 (6.2, 84.7)
ALL (%)	215 (85.7)
AML (%)	36 (14.3)
**Parent Characteristics**	
Primary caregiver	
Both mother and father^b^	169 (67.3)
^ ^Mother only	61 (24.3)
Father only	6 (2.4)
Mother works (%)	63 (25.1)
Father works (%)	172 (68.5)
Primary caregiver mother education^b^ (n = 230)	
Advanced (%)	32 (13.9)
High school (%)	39 (17.0)
Secondary school (%)	48 (20.9)
Primary school (%)	83 (36.1)
Illiterate (%)	28 (12.2)
Primary caregiver father education^b^ (n = 175)	
Advanced school (%)	22 (12.6)
High school (%)	26 (14.9)
Secondary (%)	40 (22.9)
Primary school (%)	70 (40.0)
Illiterate (%)	17 (9.7)
**Household Characteristics**	
Median number of children (IQR)	1.0 (1.0, 2.0)
Annual household income < $2000 US (%)	89 (35.5)
Access to phone (%) (n = 244)	241 (98.8)
No clean water at home (%)	108 (43.0)
No toilet at home (%)	128 (51.0)
Public transportation (bus or taxi) (%)	212 (84.5)
Median travel time (hours) from home to Hospital Bloom (IQR)	2.5 (1.5, 3.5)

a 32 children excluded because <2 years of age and missing in 1 child;

b For 169 children, both mother and father were primary caregivers. Abbreviations: IQR, interquartile range; BMI, body mass index; ALL, acute lymphoblastic leukemia; AML, acute myeloid leukemia.

### Procedure

We interviewed parents/caregivers within one month of diagnosis to establish baseline demographic and treatment variables, and to determine knowledge related to causes of fever, likelihood of having difficulty bringing their child to hospital in the event of fever, and perceived barriers to bringing their child to hospital. Literacy was measured by asking parents to describe their highest level of education completion: (a) Advanced/university/professional school; (b) High school; (c) Secondary/middle school; (d) Primary/elementary school; or (e) Illiterate. Those who self-classified themselves as illiterate were compared to those who had had completed at least primary or elementary school. In order to determine knowledge related to causes of fever, the following categories were provided and the respondent could choose all that applied: (a) Being around sick people; (b) Weather conditions; (c) Food; (d) Receiving chemotherapy; and (e) Low blood counts. Respondents were also allowed to select “other” and to provide further description.

**Table 2:** Knowledge and Barriers to Bringing Child to Hospital if Fever (N = 251).

Characteristic	ALL N = 215	AML N = 36	Entire Cohort N = 251
What causes fever?^a^			
Being around sick people	3 (1.4)	1 (2.9)	4 (1.6)
Weather conditions	21 (9.8)	1 (2.9)	22 (8.8)
Food	16 (7.4)	4 (11)	20 (8.0)
Receiving chemotherapy	11 (5.1)	0 (0.0)	11 (4.4)
Low blood counts	29 (13)	3 (8.6)	32 (13)
Infection	139 (65)	21 (60)	160 (64)
How often do you have trouble bringing your child to hospital?			
Never	87 (40)	18 (50)	105 (42)
Rarely	33 (15)	9 (25)	42 (17)
Sometimes	88 (41)	9 (25)	97 (39)
Almost always	7 (3.3)	0	7 (2.8)
Always	0	0	0
What are reasons you don’t call or go to the hospital if your child has fever andunknown counts?			
No way to get to the hospital	74 (34)	9 (25)	83 (33)
Child looks fine, no need to go	2 (0.9)	0	2 (0.8)
It doesn’t matter (it’s up to God)	0	0	0
What are barriers to bringing your child to hospital?^a^			
No one to watch other kids	9 (4.2)	1 (2.8)	10 (4.0)
Not enough money to travel	47 (21.9)	8 (22.2)	55 (22)
Cannot take time off work	2 (0.9)	0	2 (0.8)
Hospital too far away	2 (0.9)	0	2 (0.8)

a Patients could select more than one.

Abbreviations: ALL, acute lymphoblastic leukemia; AML, acute myeloid leukemia.

Interviews were repeated at the onset of each new febrile episode irrespective of whether the child was an inpatient or an outpatient. Fever was defined as an oral temperature ≥38.3°C once or ≥38.0°C twice within twelve hours. At these interviews, times from initial fever to decision to seek medical care, presentation to the hospital, obtaining a complete blood count, and initial administration of intravenous broad-spectrum antibiotic therapy were obtained for outpatients, and time from initial fever to administration of antibiotic therapy was obtained for inpatients. The ability to monitor temperature at home and use of oral antibiotic therapy at home prior to travel to the hospital were also collected. Potential causes of delay at each of these steps were solicited using open-ended questions. All interviews were conducted orally in Spanish by a single investigator who worked as a physician at Hospital Bloom (RG). Only the first febrile episode during a single hospital admission was captured but a child could have multiple febrile episodes over the course of treatment.

**Table 3:** Characteristics of the Febrile Episode Stratified by Inpatient Versus Outpatient Status (N = 379).

Characteristic	Inpatients N = 110	Outpatients N = 269
**Characteristics at Onset of Episode**		
Median days from leukemia diagnosis (IQR)	0.5 (−4.0, 70.0)	162.0 (69.0, 356.0)
Median maximum temperature in °C (IQR)	39.3 (38.8, 39.5)	39.3 (38.7, 39.5)
Median white blood cell count x10^9^ (IQR)	2.0 (0.9, 4.8)	2.0 (0.8, 4.6)
Absolute neutrophil count <0.5×10^9^ (%)	67 (60.9)	126 (46.8)
Central venous line present (%)	42 (38.2)	28 (10.4)
**Fever Practices at Home**		
Family does not own a thermometer (%)	77 (70.0)	120 (44.6)
Phone contact with healthcare professional because of fever (%)		54 (22.2)
Antibiotics taken at home before hospital (%)		18 (6.7)
**Timeline from Fever to Antibiotic Administration**		
Median hours from fever onset to hospital visit (IQR)^a^		12.5 (6.0, 24.0)
Median hours from fever onset to decision to seek medical care (IQR)^b^		10.0 (5.0, 20.0)
Median hours from decision to seek medical care to hospital visit (IQR)^c^		1.8 (1.0, 3.0)
Median hours from hospital visit to intravenous antibiotic administration (IQR)^d^		3.5 (2.2, 5.5)
Median hours from hospital visit to obtaining complete blood count (IQR)^e^		1.0 (0.5, 2.7)
Median hours from fever onset to intravenous antibiotic administration (IQR)^f^	2.0 (0.8, 5.0)	16.0 (8.3, 26.0)
**Episode Outcomes**		
Microbiologically documented infection (%)	19 (17.3)	19 (7.1)
Clinically documented infection (%)	19 (17.3)	89 (33.1)
Sepsis (%)	24 (21.8)	47 (17.5)
Infection-related mortality (%)	4 (3.6)	7 (2.6)
Intensive care unit (%)	14 (12.7)	21 (7.8)

Missing: ^a^n = 18, ^b^n = 24, ^c^n = 25, ^d^n = 28, ^e^n = 20, ^f^n = 3 for inpatients and n = 13 for outpatients.

Abbreviation: IQR – interquartile range.

Patients were monitored for febrile episodes from diagnosis until the patient recovered from their last cycle of chemotherapy, died in remission, relapsed, abandoned therapy, or experienced a second malignancy (whichever occurred first).

**Figure 1:**
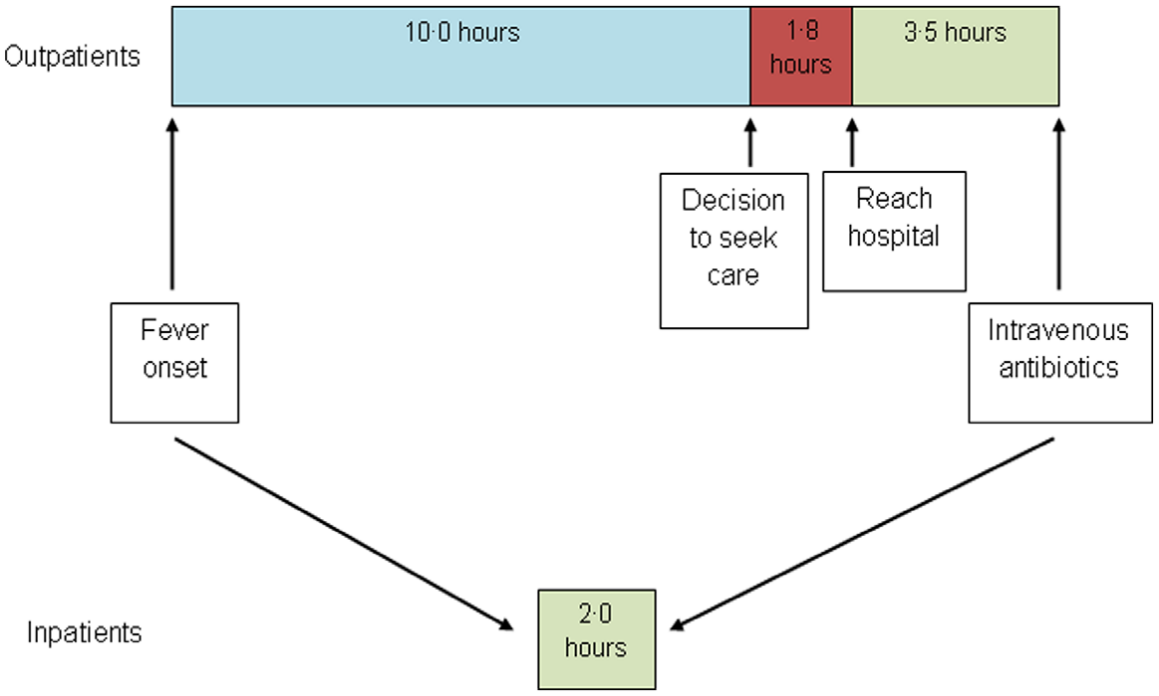
Median times from fever onset to receipt of intravenous antibiotics among outpatients and inpatients.

### Local Standards of Care

Outpatients with fever who might have been neutropenic were instructed to call or go to Hospital Bloom as soon as possible and empiric broad-spectrum intravenous antibiotics were initiated in the emergency department or oncology ward after appropriate cultures were obtained. Antibiotic prophylaxis was not uniformly administered other than for patients with AML who received prophylactic vancomycin and ciprofloxacin starting in January 2008.

**Table 4:** Factors Associated with Longer Time from Initial Fever to Decision to Seek Medical Care and from Decision to Seek Care to First Hospital Visit among Outpatient Episodes (N = 269).

Characteristic	Hours from Fever Onset to Decision to Seek Care*			Hours From Decision to Seek Care to Reach Hospital**		
	β	SE	*P Value*	β	SE	*P Value*
**Child/Household Characteristics**						
Child Male	3.84	3.16	0.227	0.24	0.22	0.266
Child Age	0.04	0.46	0.938	−0.02	0.03	0.625
AML vs. ALL	−2.34	8.31	0.779	−0.10	0.59	0.870
Mother Illiterate	11.55	5.23	0.029	0.001	0.37	0.998
Father Illiterate	15.54	6.27	0.015	−0.02	0.44	0.956
Annual Household Income < $2000	2.62	3.45	0.450	0.33	0.24	0.175
No Clean Water at Home	−1.66	3.20	0.605	0.65	0.22	0.003
No Toilet at Home	0.42	3.16	0.894	0.67	0.21	0.002
Public Transportation (bus or taxi)	5.87	4.08	0.153	0.27	0.29	0.346
Travel time to Hospital Bloom (hours)	1.01	1.09	0.356	0.30	0.07	<0.0001
**Characteristics at Episode Onset**						
Maximum Temperature (per °C)	−6.08	1.44	<.0001	0.12	0.09	0.192
Neutropenia (ANC <0.5×10^9^)	1.42	2.93	0.630	−0.04	0.18	0.816
Central Venous Line Present	0.91	4.74	0.848	−0.02	0.29	0.937
Family Does Not Own a Thermometer	3.26	3.00	0.280	0.18	0.19	0.358
**Knowledge and Barriers**						
Causes of Fever						
Weather conditions	−1.37	4.74	0.773	−0.25	0.34	0.455
Food	−7.68	6.08	0.209	−0.33	0.42	0.428
Don’t Go to Hospital Because No Way to Get to Hospital	2.14	3.26	0.513	0.28	0.23	0.220
Have Trouble Bringing Child to Hospital at least Sometimes	4.89	3.17	0.126	0.61	0.22	0.005
Barrier to Bringing Child - Money	−1.31	3.89	0.735	0.91	0.26	0.001

Abbreviations: ALL - acute lymphoblastic leukemia; AML – acute myeloid leukemia; ANC – absolute neutrophil count.

* Time Fever to Seek Care  =  time from fever onset to decision to seek care.

** Time Seek Care to Hospital  =  time from decision to seek care to reach Hospital Bloom.

In terms of chemotherapy, patients with ALL diagnosed before October 2008 were treated according to the El Salvador-Guatemala-Honduras II protocol, which was based on the St Jude Total XIII [4] and Total XV [5] protocols. Modifications included the use of only two risk groups (lower and higher), the omission of etoposide, and the administration of high dose methotrexate as a 3-hour infusion at a dose of 2 g/m^2^ for lower-risk and 3 g/m^2^ for higher-risk patients. After October 2008, patients were treated with AHOPCA-LLA 2008 which was based on ALL IC-BFM 2002. [6] Modifications included anthracycline omission for standard-risk patients, and administration of high dose methotrexate as a 4-hour infusion at a dose of 2 g/m^2^ for standard- and intermediate-risk patients, and 5 g/m^2^ for high-risk patients.

**Table 5:** Factors Associated with Sepsis and Infectious Deaths among Outpatient Episodes (N = 269).

Characteristic	Sepsis (n = 47)			Infection Death (n = 7)*		
	OR	95% CI	*P Value*	OR	95% CI	*P Value*
**Child/Household Characteristics**						
Child Male	0.95	0.47, 1.90	0.875	0.43	0.08, 2.27	0.317
Child Age	0.98	0.89, 1.09	0.742	0.97	0.71, 1.32	0.828
AML vs. ALL	2.09	0.57, 7.72	0.267	4.69	0.38, 58.26	0.230
Mother Illiterate	3.06	1.09, 8.63	0.034	3.63	0.73, 17.95	0.114
Father Illiterate	0.54	0.13, 2.18	0.384	2.26	0.24, 21.72	0.480
Annual Household Income < $2000	1.02	0.45, 2.31	0.966	13.90	1.62, 119.10	0.016
No Clean Water at Home	0.69	0.34, 1.43	0.318	1.76	0.38,8.12	0.470
No Toilet at Home	1.00	0.50, 1.98	0.992	2.05	0.38, 10.95	0.402
Public Transportation (bus or taxi)	1.41	0.53, 3.73	0.488	**		
Travel time to Hospital Bloom (hours)	1.07	0.90, 1.27	0.461	1.36	1.03, 1.81	0.031
**Characteristics at Episode Onset**						
Maximum Temperature in °C	1.45	0.86, 2.45	0.159	0.57	0.23, 1.42	0.229
Neutropenia (ANC <0.5×10^9^)	1.86	0.96, 3.63	0.067	2.91	0.56, 15.12	0.203
Central Venous Line Present	2.53	1.08, 5.94	0.033	**		
Family Does Not Own a Thermometer	0.90	0.46, 1.80	0.772	1.68	0.37, 7.68	0.505
**Knowledge and Barriers**						
Causes of Fever						
Weather conditions	2.64	1.06, 6.62	0.038	1.16	0.16, 8.42	0.886
Food	2.02	0.45, 8.98	0.356	5.12	1.08, 24.28	0.040
Don’t Go to Hospital Because No Way to Get to Hospital	0.73	0.35, 1.51	0.394	0.65	0.12, 3.49	0.614
Have Trouble Bringing Child to Hospital at least Sometimes	1.04	0.51, 2.11	0.924	0.87	0.19, 4.02	0.863
Barrier to Bringing Child - Money	1.24	0.55, 2.77	0.607	1.58	0.29, 8.73	0.602
**Times to Assessment and Treatment**						
Hours fever onset to decision to seek medical care	1.01	0.99, 1.02	0.495	1.00	0.95, 1.05	0.925
Hours from decision to seek medical care to hospital visit	1.01	0.84, 1.23	0.890	0.74	0.38, 1.44	0.375
Hours from hospital visit to intravenous antibiotics	0.79	0.63, 0.99	0.041	1.02	0.80, 1.28	0.889

Abbreviations: ALL - acute lymphoblastic leukemia; AML – acute myeloid leukemia; ANC – absolute neutrophil count; OR – odds ratio.

* These results should be viewed very cautiously as there were only 7 infectious deaths among outpatients.

** OR not estimable because all 7 infectious deaths occurred in children taking public transportation and in those who did not have a central venous line.

Patients with AML were treated with the AHOPCA-AML 2007 protocol which was based on NOPHO-AML 93 [7] but with induction therapy according to BFM-AML 93. [8] Modifications included the treatment of all patients without stem cell transplantation, triple intrathecal therapy in each block of treatment and induction therapy with 8 days of cytarabine, 3 days of daunorubicin reduced by 66% and 3 days of etoposide (ADE). Patients with less than 5% bone marrow blasts at day 29 received a second course of ADE after hematological recovery. In April 2010, daunorubicin induction was reduced to 50% of the original BFM-AML 93 protocol, [8] etoposide was removed, and the fourth course of consolidation therapy was omitted for patients who had good response to the first induction treatment. Patients with acute promyelocytic leukemia (APL) were treated with AHOPCA-APL 2008, which was based on European APL 91 [9].

**Figure 2:**
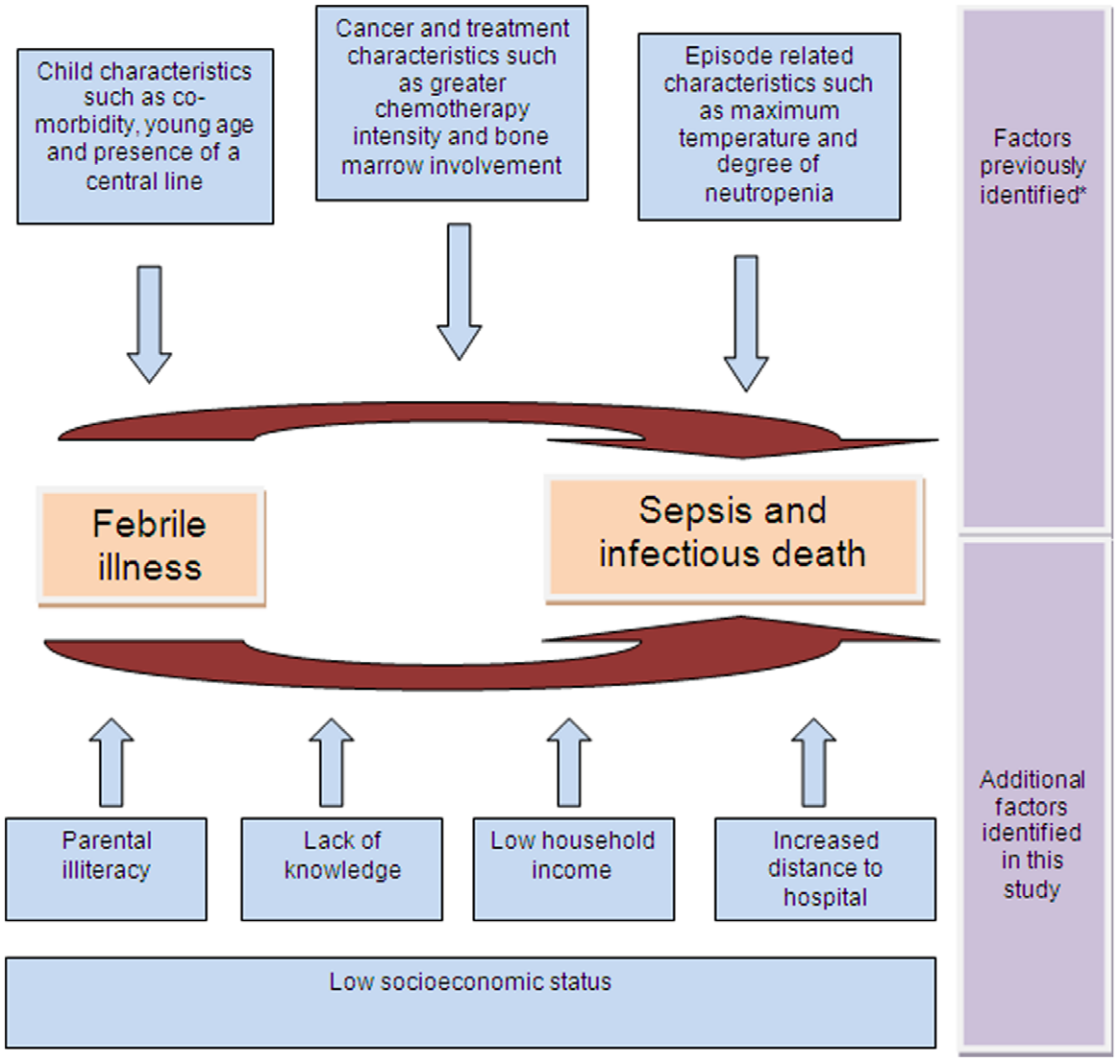
Factors associated with sepsis and infectious deaths in pediatric acute leukemia in low/middle income countries*. Based on a systematic review in pediatric oncology. Factors listed are illustrative and not meant to be exhaustive. [20].

### Outcomes

Sepsis was defined as systemic inflammatory response syndrome in the presence of suspected or proven infection and organ dysfunction according to international consensus guidelines. [10], [11] Invasive infection was defined as the occurrence of one or more cultures positive for a pathogen obtained from a usually sterile site. However, positive cultures with common contaminants, such as coagulase negative *Staphylococcus,* required two positive cultures for the same organism or occurrence of sepsis to be considered a true infection [12], [13].

### Potential Predictors

Potential predictors of outcomes were categorized as demographic information of the child, parent and household; characteristics at episode onset; and knowledge and barriers as measured at the baseline (first) interview that was conducted within one month of diagnosis. Child demographics were gender, age, and diagnosis (AML vs. ALL). Parental education was dichotomized as illiterate compared with at least primary school education. Household features included ≥ or < US$2000 annual household income, which was chosen *a priori* based upon our previous studies. [2], [14] Other household features evaluated included the availability of clean water and a toilet at home, method of transportation (public vs. private) and travel time in hours to Hospital Bloom. Features recorded at the onset of each febrile episode included maximum temperature, neutropenia, presence of a central venous line and whether the family had a thermometer at home. Items included in regression modeling from the survey of knowledge and barriers were: belief that fever was caused by weather or food; not going to the hospital if fever develops because lacked means to travel; have trouble bringing child to Hospital Bloom at least sometimes (on a five-point Likert scale where 1 = never and 5 = always have trouble); and lack of money is a barrier to bringing the child to hospital.

For the outcomes of sepsis and infectious deaths, we also included three time intervals as potential predictors for outpatients: hours from fever onset to decision to seek medical care; hours from decision to seek care to hospital visit; and hours from hospital visit to intravenous antibiotics.

### Statistics

Given the fundamental differences expected between inpatients and outpatients, all analyses were stratified by location at onset of fever. In order to identify factors associated with times to presentation and treatment, we conducted a repeated measures linear regression using Proc Mixed in SAS. Determination of factors associated with sepsis and infectious mortality were conducted with repeated logistic regression analysis using generalized estimating equations. These approaches were used in order to account for each child contributing multiple episodes and to adjust for any potential correlation between episodes within an individual child. Multiple regression was planned for sepsis and infectious mortality including variables significant in univariate analysis. All tests of significance were two-sided, and statistical significance was defined as P<0.05. Statistical analyses were performed using the SAS statistical program (SAS-PC, version 9.3; SAS Institute Inc., Cary, NC).

## Results

Between May 1, 2008 and March 31, 2011, 254 potentially eligible children with acute leukemia presented to Hospital Bloom. One was not approached as the patient was unwell and died before consent could be obtained, one did not have a competent legal guardian and one refused participation; thus, 251 children with *de novo* ALL and AML were enrolled. Child, parent and household characteristics are illustrated in Table 1. Most children had ALL (215, 85.7%) and among these children, 175 (81.4%) were treated with AHOPCA LLA 2008. Among the children with AML, 30 (83.3%) were treated with AHOPCA LMA 2007.

Table 2 illustrates knowledge and barriers related to fever care among parent respondents; these responses were obtained at the initial interview within one month of diagnosis. Most parents knew that infection can cause fever although 8.8% thought that fever could be caused by the weather and 8.0% thought that fever could be caused by certain foods. Eight-five participants selected “other” as a cause of fever, and listed leukemia (n = 47), trauma (n = 2), allergies (n = 4) insect bites (n = 2), pollution (n = 3), dust, dirt or contamination (n = 10), and miscellaneous/not specified (n = 17) as the potential cause. In total, 104 (41.4%) said that they sometimes, almost always or always had difficulty travelling to the hospital if their child had fever. The main reason that parents would not go to the hospital if their child had fever was because they had no way to travel to the hospital. The most common barrier to bringing their child to hospital was lack of money.

During the study period, 47 (18.7%) relapsed and 18 (7.2%) abandoned therapy during the observation period and thus, were no longer observed for infection outcomes after those events. There were 379 febrile episodes that occurred during treatment; 110 in inpatients and 269 in outpatients. Table 3 illustrates characteristics at the onset of the episode, fever practices at home, times to seek care, presentation to hospital and receipt of intravenous antibiotics (Figure 1) and episode outcomes. Among outpatients, the major source of delay is time from fever onset to decision to seek care with 75% of participants waiting for 5 hours or more. Once the decision to seek care had been made, the median time to reach the hospital was 1.8 hours. In total, there were 71 (18.7%) episodes of sepsis and 11 (2.9%) infectious deaths.

Table 4 illustrates factors associated with longer time from initial fever to decision to seek medical care and time from decision to seek care to Hospital Bloom among outpatient episodes. Parents who were illiterate had a 12 to 16 hour delay in deciding to seek medical care compared to parents who were literate. Once the decision to seek medical care had been made, parental literacy did not influence time to first hospital visit. Rather, anticipated travel time to Hospital Bloom, lack of clean water and no toilet at home were associated with longer time to reach hospital. Parents who stated that they had trouble travelling to the hospital and that lack of money was a barrier to travel had a greater delay in reaching the hospital. Table S1 describes factors associated with longer time from first hospital visit to intravenous antibiotics among outpatient and from fever to intravenous antibiotics among inpatient episodes. Among inpatients, those with AML had shorter time to first antibiotics.

Table 5 illustrates factors associated with sepsis and infectious deaths among outpatient episodes. The following factors were significantly associated with sepsis: maternal illiteracy (OR 3.06, 95% CI 1.09 to 8.63; P = 0.034), central line (OR 2.53, 95% CI 1.08 to 5.94; P = 0.033), and belief about weather as a cause of fever (OR 2.64, 95% CI 1.06 to 6.62; P = 0.038). The following factors were significantly associated with infectious death: annual household income <US$2000 (OR 13.90, 95% CI 1.62 to 119.10; P = 0.016), anticipated travel time to Hospital Bloom (OR 1.36, 95% CI 1.03 to 1.81; P = 0.031) and belief about food as a cause of fever (OR 5.12, 95% CI 1.08 to 24.28; P = 0.040). Time to reach hospital was not predictive of sepsis or infectious death.

In the multiple regression analysis of sepsis, maternal illiteracy, presence of a central line and belief that weather causes fever were examined. Each factor was independently associated with sepsis: maternal illiteracy (OR 3.17, 95% CI 1.24 to 8.11; P = 0.016), central line (OR 2.88, 95% CI 1.25 to 6.64; P = 0.013), and belief about weather as a cause of fever (OR 2.67, 95% CI 1.22 to 5.85; P = 0.014). Time from hospital visit to antibiotic administration was not included since shorter time intervals were most likely due to sepsis at the time of presentation rather than a cause of sepsis. Multiple regression was not conducted for infection-related mortality because there were only 7 events.

Table S2 describes factors associated with sepsis among inpatients. Higher maximum temperature and neutropenia were significant predictors of sepsis in the inpatient setting whereas time to antibiotic initiation was not predictive. Analysis of infectious deaths was not presented since there were only 4 inpatient deaths. Figure 2 summarizes how the findings from this study contribute to our understanding of risk factors for sepsis and infectious deaths in children with acute leukemia in LMIC.

The qualitative comments reflected similar findings. Parents identified lacking transportation, delays during travel (for example transit strikes and inclement weather) and long emergency room wait times as reasons for longer times to antibiotic treatment.

## Discussion

In this prospective study of children with acute leukemia in El Salvador, we found that parental illiteracy was associated with delays in deciding to seek care for fever. We also identified those with longer travel time to hospital once the decision to seek care had been made; these were children who had an anticipated longer travel time to Hospital Bloom, parental anticipation of having difficulty bringing their child to hospital, and parental perception that lack of money is a barrier to travel. Together, these findings suggest that poor socioeconomic status influences delays in deciding to seek care and reaching the hospital for febrile children with leukemia.

We also found that maternal illiteracy was associated with sepsis while low household income and longer anticipated travel time to Hospital Bloom were associated with infectious mortality. These findings similarly suggest that poor socioeconomic status influences severe outcomes of fever in children with leukemia. An association between illiteracy and poor health outcomes has been demonstrated in other clinical settings and in particular, lack of literacy has been associated with poor compliance in a variety of clinical conditions[15]–[18] and worse outcomes. [19] However, our study suggests that one important mechanism by which illiteracy and poverty may worsen infection outcomes in LMIC is by delaying time to treatment for fever.

While low socioeconomic status was associated with delays in times to treatment for fever and with severe fever outcomes, time from fever to hospital presentation was not associated with sepsis or infectious mortality directly. It is possible that delays in receiving treatment for fever are not associated with sepsis or infectious mortality. More likely, the relationship between socioeconomic status, delays to treatment and fever outcomes is not linear but rather, complex and confounded by multiple factors. An important confounder that we could not measure is infection severity. For example, from the current analysis, those with sepsis had a shorter time to first antibiotics once they had reached the hospital. By analogy, children who were more ill may have had parents who decided to seek medical care earlier and who reached hospital sooner. Consequently, it is not surprising that we failed to show an association between time to hospital and sepsis or mortality.

This study is important because we provide one example of how to assess barriers to health in children with severe/chronic conditions in LMIC. Most children with cancer live in LMIC and interventions that can improve survival for these children will be impactful. Interventions should be considered that focus in at least two different directions. First, our findings suggests that targeted educational strategies for illiterate parents to seek care early and to define ahead of time the plan for transportation to the hospital should fever occur could reduce death from infection in LMIC. Provision of accommodation such as a nearby guest house during periods of neutropenia for the highest risk children should also be considered. Second, interventions such as antibiotic prophylaxis or oral antibiotics while travelling to the hospital may be considered although the impact on antibiotic resistance is an important issue.

A major strength of our study was the use of a prospective observational design to collect information at multiple time points; such a design was critical to achieving our objectives. A second strength was the recruitment of large numbers of children with acute leukemia with similar anti-cancer treatment. However, our results must be interpreted in light of its limitations. Our study is limited by its observational nature and there are many potential confounders that could not be measured such as infection severity at the onset of fever. Second, it is not clear how generalizable these findings are outside of El Salvador. However, we have no *a priori* reason to believe that findings would be substantially different in other nations with similar healthcare systems and economics. Finally, there were 7 outpatient infectious deaths and thus, the power of this analysis was limited.

In conclusion, illiteracy and poverty are associated with delays in treatment of fever and with sepsis and infectious mortality in pediatric leukemia in the LMIC of El Salvador. Future work should focus on reducing times to treatment for febrile children and identifying strategies to decrease sepsis and infectious mortality that target children of low socioeconomic status.

## Supporting Information

Table S1 **Factors Associated with Longer Time from First Hospital Visit to Intravenous Antibiotics Among Outpatient Episodes and from Fever to Intravenous Antibiotics Among Inpatient Episodes.**
(DOC)

Table S2 **Factors Associated with Sepsis among Inpatient Episodes (N = 110).**
(DOC)
