# Supplementary material for: Low Socioeconomic Status Is Associated with Prolonged Times to Assessment and Treatment, Sepsis and Infectious Death in Pediatric Fever in El Salvador
Source: PLoS One. 2012 Aug 22;7(8):e43639. doi: 10.1371/journal.pone.0043639 (PMC3425537; doi:10.1371/journal.pone.0043639)
Supplement: Table S1 — Factors Associated with Longer Time from First Hospital Visit to Intravenous Antibiotics Among Outpatient Episodes and from Fever to Intravenous Antibiotics Among Inpatient Episodes. (DOC) [file pone.0043639.s001.doc]

**Table S1. Factors Associated with Longer Time from First Hospital Visit to Intravenous Antibiotics Among Outpatient Episodes and from Fever to Intravenous Antibiotics Among Inpatient Episodes**

| Characteristic | Outpatient (n=269) | | | Inpatient (N=110) | | |
| --- | --- | --- | --- | --- | --- | --- |
|  | β | SE | *P Value* | β | SE | *P Value* |
| **Child/ Household Characteristics** |  |  |  |  |  |  |
| Child Male | 0.34 | 0.41 | 0.414 | -0.59 | 1.07 | 0.587 |
| Child Age | 0.03 | 0.06 | 0.685 | -0.04 | 0.13 | 0.736 |
| AML vs. ALL | 1.98 | 1.08 | 0.070 | -3.43 | 1.02 | 0.002 |
| Mother Illiterate | 1.18 | 0.68 | 0.087 | 0.17 | 1.59 | 0.916 |
| Father Illiterate | 0.92 | 0.80 | 0.253 | -2.45 | 2.81 | 0.391 |
| Annual Household Income < $2000 | -0.05 | 0.45 | 0.916 | -1.64 | 1.11 | 0.150 |
| No Clean Water at Home | 0.65 | 0.42 | 0.120 | -0.12 | 1.08 | 0.909 |
| No Toilet at Home | 0.94 | 0.41 | 0.024 | 0.42 | 1.08 | 0.702 |
| Public Transportation (bus or taxi) | 0.19 | 0.55 | 0.734 | -1.18 | 1.63 | 0.475 |
| Travel time to Hospital Bloom (hours) | -0.05 | 0.14 | 0.717 | -2.40 | 0.33 | 0.476 |
|  |  |  |  |  |  |  |
| **Characteristics at Episode Onset** |  |  |  |  |  |  |
| Maximum Temperature in °C | -0.13 | 0.22 | 0.542 | 0.01 | 0.70 | 0.985 |
| Neutropenia (ANC <0.5 x10^9^) | 0.22 | 0.41 | 0.588 | -0.35 | 1.10 | 0.751 |
| Central Venous Line Present | 0.73 | 0.68 | 0.284 | -0.78 | 1.10 | 0.483 |
| Family Does Not Own a Thermometer | 0.40 | 0.42 | 0.340 | -1.17 | 1.16 | 0.322 |
|  |  |  |  |  |  |  |
| **Knowledge and Barriers** |  |  |  |  |  |  |
| Causes of Fever |  |  |  |  |  |  |
| Weather conditions | 0.24 | 0.61 | 0.689 | -1.75 | 2.33 | 0.459 |
| Food | 0.80 | 0.78 | 0.307 | 1.56 | 2.03 | 0.450 |
| Don’t Go to Hospital Because No Way to Get to Hospital | -0.29 | 0.43 | 0.496 | 1.07 | 1.16 | 0.364 |
| Have Trouble Bringing Child to Hospital at least Sometimes | -0.02 | 0.42 | 0.964 | 0.003 | 1.13 | 0.998 |
| Barrier to Bringing Child - Money | -0.25 | 0.51 | 0.621 | -1.89 | 1.39 | 0.185 |

Abbreviations: ALL - acute lymphoblastic leukemia; AML – acute myeloid leukemia; ANC – absolute neutrophil count.
