# Supplementary material for: Low Socioeconomic Status Is Associated with Prolonged Times to Assessment and Treatment, Sepsis and Infectious Death in Pediatric Fever in El Salvador
Source: PLoS One. 2012 Aug 22;7(8):e43639. doi: 10.1371/journal.pone.0043639 (PMC3425537; doi:10.1371/journal.pone.0043639)
Supplement: Table S2 — Factors Associated with Sepsis among Inpatient Episodes (N = 110). (DOC) [file pone.0043639.s002.doc]

**Table S2. Factors Associated with Sepsis among Inpatient Episodes (N=110)**

| Characteristic | Sepsis (n=24) | | |
| --- | --- | --- | --- |
|  | OR | 95% CI | *P value* |
| **Child/ Household Characteristics** |  |  |  |
| Child Male | 0.65 | 0.27, 1.57 | 0.340 |
| Child Age | 0.93 | 0.82, 1.06 | 0.292 |
| AML vs. ALL | 1.24 | 0.51, 2.98 | 0.634 |
|  |  |  |  |
| **Characteristics at Episode Onset** |  |  |  |
| Maximum Temperature in °C | 2.40 | 1.19, 4.87 | 0.015 |
| Neutropenia (ANC <0.5 x10^9^) | 6.09 | 1.72, 21.58 | 0.005 |
| Central Venous Line Present | 1.87 | 0.77, 4.50 | 0.165 |
|  |  |  |  |
| **Times to Assessment and Treatment** |  |  |  |
| Hours from fever to intravenous antibiotics | 0.98 | 0.88, 1.08 | 0.651 |

Abbreviations: ALL - acute lymphoblastic leukemia; AML – acute myeloid leukemia; ANC – absolute neutrophil count; OR – odds ratio.
